# Medical Society of the County of Albany

**Published:** 1873-02

**Authors:** F. C. Curtis

**Affiliations:** Secretary


ART. II.—*Medical Society of the County of Albany. Semi-Monthly Meeting. January* 22, 1873.

Reported by F. C. CURTIS, M. D., Secretary.

Dr. ALBERT VAN DERVEER, President, in the Chair.

Appropriate resolutions were offered by the committee appointed at a previous meeting in relation to the death of Dr. J. F. Crounse.

The names of the following gentlemen were proposed for membership: Drs. John U. Haynes, James S. Hill, D. L. Dayton and E. A. Green. They were referred to the Board of Censors.

Dr. WILLIAM HAILES spoke on the subject of *The Anatomy of Necrosis and the process of repair*, illustrating the subject by means of the Stereopticon.*

The general plan of his remarks was first to treat of Necrosis or death as it occurs in connective tissue, and to compare the processes which nature adopts in dealing with the same affection in

* It may be remarked that this method of illustrating various subjects, more particularly the minute structure of pathological and normal tissues, has been made good use of in the Medical College, by Professor Van Derveer especially. Nothing could be more perfectly adapted than this to the purpose of teaching microscopical appearances to a large class, the field being thrown upon a screen, greatly enlarged, and visible to all at once. It is novel in this use I believe, and deserves to be more widely employed. F. C. C.

the more compact and unyielding tissues, as the osseous and tendinous.

The subject he illustrated by a series of diagrams of the microscopical appearances of normal and inflamed bone, and a large number of photographic slides made directly from pathological specimens in the museum of the Albany Medical College. These slides or transparencies were enlarged many diameters—thrown upon a white wall by means of the oxy-calcium lantern. The College museum is extremely rich in the variety and number of its specimens, being one of the finest collections in the State.

The mode of separation of the sequestrum, the formation of the involucrum, the presence of the living wall of granulation tissue between the septic elements of decaying tissues and the open mouths of absorbent vessels, and the almost complete analogy existing between the various structures in accomplishing the separation of dead parts and the reproduction of the new were spoken of at length.

The microscopic and pathological anatomy of the subject was fully illustrated. The minute structure of the parts at the different stages of the affection, and the appearance of actual specimens in the various phases of necrosis were exhibited. The modifications of the vascular supply in different tissues, and their various powers of anastomosis were fully discussed.

At the conclusion of the paper a vote of thanks was given to Dr. Hailes for the effort he had made to entertain the society.

After a recess and refreshments Dr. E. H. Davis read a paper entitled *Pleuritic Effusions Treated by Paracentesis.* After a few peliminary remarks he continued: In cases of effusions into the cavity of the chest it is important to determine as nearly as possible the amount and character of the fluid. If there be any doubt as to the nature of the case, after having examined the patient in the ordinary manner, we can in most instances make use of the exploring needle with perfect safety, to correct an error or confirm our diagnosis.

Having determined the existence of fluid in the pleural sac, and that an opening into that cavity for its evacuation is necessary, three pertinent questions naturally arise—when, where and how shall we operate? Not that in all cases operation is necessary, for if the fluid is serum simply, a reasonable hope may be indulged that under favorable circumstances it will be absorbed by the aid of appropriate remedies, however large the quantity. But, if the sac contains pus, constituting empyæma, the hope of absorption is greatly diminished, if in fact it is not entirely precluded. Therapeutic agents have but little influence in promoting the absorption of pus, and if it were absorbed its influence might prove only disastrous. When therefore paracentesis becomes necessary it should be resorted to without delay. The place of selection for making the opening is where we are sure we are below the level of the fluid and where we can most readily reach it. Miller's Surgery directs us to carefully avoid the margins of the ribs, but in most cases I have selected the upper margin. In some cases I have used the bistoury and in others the trocar. When there is little or no bulging of the inter-costal spaces, or where there is the least doubt as to what may be underneath, I should greatly prefer the bistoury, so as to make an open wound and penetrate the cavity of the chest no further than barely sufficient to divide the pleura. In other cases after dividing the integument the trocar is all that is necessary and very convenient.

The following cases are presented: J. K., a mechanic, aged 17, while on his way to this city on a canal boat in the month of June, was taken violently sick with pleuro-pneumonia. I was called to attend him about ten days after the onset and found him still laboring under severe inflammatory action, which, after continuing ten or twelve days more, began slowly to abate and gave some encouragement of recovery. But the disease still continued in a subacute form, and after a few weeks he began to show symptoms of purulent effusion into the left pleural cavity. This gradually increased until the fifth of November, when I opened the chest for its removal. Previous to the operation the left side of the chest was greatly disturbed, and the intercostal space, prominent. The whole of that side from the sternum to the spine was without rale or respiratory murmur. He had frequent cough with but little expectoration, and also dyspnœa. Appetite and digestion were greatly impaired, with rapidly increasing emaciation.

For the operation I had him taken from his bed and seated in a common chair reversed, with his head and arms resting upon its back. I divided the integument by puncturing it and cutting from within outwards at a point about four inches to the left of the spine, and over the lower margin of the eighth rib. I then pressed the integument upwards and passed a trocar through the wall close to the upper margin of the same rib, drawing off ten pints of thin pus. As he now became faint I stopped the discharge with a tent and dressed the wound with adhesive plaster, with a bandage around the body. The next day I drew off five pints and the day following as much more, for the first time emptying the cavity. After this the tent was removed daily and the accumulated pus permitted to escape. The amount discharged during the first ten days was estimated by a friend of the family at thirty-two pints. He now gradually improved, and his appetite was good so that he took a large amount of food which was well digested. For five months he continued to gain and was able to walk about the house, sometimes remaining up during the entire day. But in the month of April his strength began to fail and he died about the first of May. A post mortem examination was not allowed.

Two prominent points of interest in the case are: 1st, the large amount of pus discharged, and 2d, the length of time the system was not only able to sustain this great drain upon its resources, but to improve in strength and flesh, being finally borne down only when returning health seemed almost in his grasp.

G. P., aged 33, captain of a canal boat; a man of full habit, and strong, vigorous constitution. He was taken sick while on the canal, and some time after I was called to visit him. I found him suffering from severe inflammation of the right lung, involving the pleura and already threatening suppuration. His previous treatment had been entirely inadequate to his disease. Symptoms, after a short time, of empyæma became evident, affecting the right side, and believing that nothing could be gained by delay I determined to evacuate the pus while his strength was good. This I did, puncturing the chest wall by slow and cautious incisions under the right axilla, close to the upper margin of the sixth rib, and drew off about five pints. I then inserted a tent and dressed with adhesive plaster with a bandage. This was removed daily and the discharge continued in gradually diminishing quantity for about eight weeks, when it entirely ceased and the wound healed. Before the operation his cough was severe and he expectorated pus freely. If at any time he lay upon his right side he could raise little or nothing, but if he turned upon his left side the flow of pus would occasionally threaten suffocation, and he would expectorate from a gill to a half pint in a few minutes, showing that the abscess had not only involved the lung, but had formed a communication with the bronchi. From this time forward he continued to improve slowly until he completely regained health. This was at the time of the uprisal of the south, and at the first call for troops at the north he enlisted in the army and served with credit through the war.

W. D., a farmer, aged 30, while plowing in the spring of the year caught a severe cold, which brought on a difficulty of the lungs from which he never entirely recovered. One year after the following January I first saw him. He had what I regarded the inflammatory phthisis of Niemeyer. The right lung was to a great extent disorganized, but the left was still in a condition to perform its function. By means of succussion, evidence was obtained that air as well as fluid was in the right pleural cavity, the splashing sound being heard even before the ear reached the chest. He suffered from dyspnœa, and was unable to lie down in bed from a sense of impending suffocation. I believed his case hopeless and stated my opinion to him. But he urged me to draw off the pus, which I told him his chest contained, with the view of relieving his difficulty of breathing. So, with the hope of affording this relief and possibly prolonging his life, I determined to remove it. At my next visit I took with me Dr. Purdy of Elmira and, after having his approval, I operated as in the first case, entering the chest cavity close to the upper margin of the rib and near the inferior angle of the scapula, drawing off seven pints of pus greatly to his relief. His laborious respiration was entirely removed and he could lie in bed with comfort, which he had not done for many weeks before. He lived only three weeks after the operation, but the relief he experienced from it was satisfactory. A post mortem examination was not permitted. There are three questions of interest connected with the case: Did this pus have its origin in a pulmonary abscess which burst into the pleural cavity? Are such large accumulations of matter liable to occur in the course of phthisis? Did the relief obtained justify the operation at so late a period?

H. B., 19 years of age, son of a farmer, was taken with severe illness, three weeks after the beginning of which I first saw him, in consultation with several other medical gentlemen. There was no room for doubt that the diagnosis of pleurisy, made by the attending physician, was correct. We found him lying in bed, fixed on his left side, unable to change his position without great inconvenience, and his countenance was expressive of anxiety and suffering. There was no respiratory sound, natural or morbid, over the whole left side. A prominence of the intercostal spaces was barely perceptible, and the side was a little distended. By placing the hand over the region of the heart I marked the distinctness with which I seemed to feel its concussion against the wall of the chest, the apex being near the margin of the right axilla. I believed the case to be one of hydrothorax and at once proposed the evacuation of the fluid by incision, but as there was a difference of opinion on the part of a distinguished physician present, it was thought best to delay. Four days after we again met, and as we were still unable to agree as to the propriety of operating, we explained the difficulty to the friends of the patient, and as he was rapidly failing they expressed a desire that an effort be made for his relief.

We accordingly had him raised to a half sitting posture in bed, his head turned to the right and his left arm moved back and out of the way, assistants holding him in this position. An exploring needle was passed through the space between the sixth and seventh ribs, three inches to the left of the sternum, and on withdrawing it water trickled down his side. With a bistoury I then made an incision two inches in length over the lower margin of the seventh rib through the integument, which was then slipped upward and I carefully divided the tissues down to the pleura by shortened incisions, puncturing the membrane with a sharp pointed bistoury. The fluid discharged with a force that carried it ten or twelve inches from his side. It continued to flow until we had a common water pail half full of a thin, slightly albuminous liquid. During the discharge the heart gradually returned from its abnormal position and the patient instead of becoming faint or exhausted, expressed himself greatly relieved. We gradually reclined him over the edge of the bed till he was placed fully on his left side, and at the first sound of ingress of air to the chest I slid the integument downward and closed the wound with adhesive plaster and a bandage. There was no return of the effusion and the wound was not reopened. He soon began to improve and continued to do so with no untoward event until he completely regained his health. The disease left him with the left side of the chest slightly contracted, but the lung was healthy and he was able to follow the laborious occupation of house carpenter.

Dr. Bigelow remarked that the paper was interesting and the cases detailed rare and unique. He thought that it deserved a fuller discussion than the lateness of the hour would allow, and moved that its discussion be postponed and made the first in order at the next meeting. Carried.

